# Maternal survival of patients with pregnancy‐associated cancers in Taiwan – A national population‐based study

**DOI:** 10.1002/cam4.3565

**Published:** 2020-10-25

**Authors:** Sin‐Syue Li, Ya‐Ting Hsu, Chih‐Chieh Yen, Ying‐Wen Chen, Pei‐Ying Wu, Kung‐Chao Chang, Chung‐Yi Li, Tsai‐Yun Chen

**Affiliations:** ^1^ Division of Hematology / Oncology, Department of Internal Medicine National Cheng Kung University Hospital, College of Medicine, National Cheng Kung University Tainan Taiwan; ^2^ Institute of Clinical Medicine, College of Medicine National Cheng Kung University Tainan Taiwan; ^3^ Department of Obstetrics & Gynecology National Cheng Kung University Hospital, College of Medicine, National Cheng Kung University Tainan Taiwan; ^4^ Department of Pathology National Cheng Kung University Hospital, College of Medicine, National Cheng Kung University Tainan Taiwan; ^5^ Department and Graduate Institute of Public Health, College of Medicine National Cheng Kung University Tainan Taiwan

**Keywords:** breast cancer, colorectal cancer, epidemiology and prevention, lymphoma, Survival, women's cancer

## Abstract

Pregnancy‐associated cancer (PAC), defined as cancers diagnosed during pregnancy or the first year after delivery, affects one to two in every 1000 pregnancies. Although PAC is expected to be a growing issue, information about PAC in the Asian population is still scarce. Women with cancer diagnosed at the age of 16–49 years between 2001 and 2015 were selected from the Taiwan Cancer Registry and linked with the National Birth Reporting Database to identify PAC patients. We compared the overall survival of patients with PAC to patients without pregnancy. Among 126,646 female cancer patients of childbearing age, 512 were diagnosed during pregnancy, and 2151 during the first postpartum year. Breast cancer was the most common PAC (*N* = 755, 28%). Compared with patients without pregnancy in the control group, patients with cancers diagnosed during pregnancy and the first postpartum year generally had more advanced stages (odds ratio 1.35 and 1.36, 95% confidence interval [CI] 1.02–1.77 and 1.18–1.57, respectively). For all cancer types combined and controlled for the stage, age, and year of diagnosis, patients with PAC had similar overall survival with those in the control group, with a hazard ratio (HR) of 1.07 (95% CI 0.80–1.41) for the pregnancy group and HR 1.02 (95% CI 0.88–1.18) for the postpartum group. The diagnosis of breast cancer during the first postpartum year was linked with shorter survival (HR 1.34, 95% CI 1.05–1.72). In contrast, patients with postpartum lymphoma (HR 0.11, 95% CI 0.02–0.79) and cervical cancer (HR 0.40, 95% CI 0.20–0.82) had better prognosis. In general, the diagnosis of cancer during pregnancy or the first postpartum year does not affect the survival of patients with most cancer types. Exceptions include the worse prognosis of postpartum breast cancer and the better outcome of postpartum lymphoma and cervical cancer.

## Graphical abstract

A national population‐based study of patients with pregnancy‐associated cancers from Taiwan showed various impacts of pregnancy on the outcome of patients with different types of cancer.

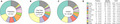

## 1 INTRODUCTION

Pregnancy‐associated cancers (PAC), usually defined as cancers diagnosed during pregnancy or the first year after delivery, had been reported to affect one to two in every 1000 pregnancies.^1^, ^2^, ^3^, ^4^, ^5^ Malignant melanoma, breast cancer, cervical cancer, lymphoma, and leukemia are among the most common PAC.^1^, ^2^, ^3^, ^5^, ^6^, ^7^

While more women in the modern world prefer delaying childbearing, PAC is expected to be a growing issue.^8^ However, the diagnosis and treatment of PAC remain challenging, and it is a concern that the patients' outcome may also be affected by the status of gestation.^9^, ^10^

The increased exposure to hormones during pregnancy and lactation may change the tumor's behavior, especially hormone‐dependent tumors like breast cancer.^11^, ^12^, ^13^ During pregnancy, the suppressed immune system and increased vascularization may potentiate the tumor aggressiveness.^14^, ^15^, ^16^, ^17^ Moreover physiological changes during pregnancy and lactation, such as increased plasma volume and glomerular filtration rate, may alter anticancer drugs' pharmacokinetics and affect the treatment outcome.^18^

Among all types of PAC, the outcome of pregnancy‐associated breast cancer is the most widely studied. Patients with breast cancers diagnosed during pregnancy are usually in more advanced stages, have larger tumor sizes, have more lymph node involvement, have more undifferentiated histology, and with poorer prognosis.^11^, ^12^, ^13^, ^25^ Furthermore, patients with postpartum breast cancer also have a poorer prognosis than patients without pregnancy, and the worse prognosis may persist longer than 5 years after childbirth.^22^, ^26^ After controlling other prognostic factors, breast cancer diagnosed during pregnancy does not confer to an adverse outcome.^12^, ^13^, ^25^ In contrast, the diagnosis of postpartum breast cancer is an independent prognostic marker shown in some studies.^3^, ^27^

Beyond breast cancer, studies regarding other PAC types are more scarce and are usually with limited case numbers. Some cancers commonly diagnosed in women of childbearing women have significant differences in epidemiologic and disease characteristics between the East and the West. For example, there is a higher proportion of younger and premenopausal breast cancer patients in Asia.^28^ Furthermore, malignant lymphoma has a lower incidence and a higher proportion of T and natural killer cell lymphomas in East Asia than in the West.^29^ Therefore, in the Asian population, a different PAC landscape is expected but was only reported in a few reports with limited case numbers.^30^, ^31^, ^32^

In this study, we report the incidence and cancer types of PAC from a national population‐based cohort of patients in Taiwan and explore the links between pregnancy status with the survival of patients with PAC.

## 2 MATERIAL AND METHODS

### 2.1 Data sources

Taiwan Cancer Registry (TCR) was founded in 1979 to collect information about cancer cases diagnosed in hospitals with more than 50 beds. TCR had more than 98% overall coverage of potential cancer patients in Taiwan. The cancer diagnosis was verified by histological or cytological exams in 93% of patients (97.6% if liver excluded).^33^ All cases were registered in the short‐form database with personal data and information about diagnosis and treatment. In addition to the essential information in the short‐form database, the long‐form database was established in 2002 to collect detailed information about staging, treatment, and recurrence of common malignancies, including cancers of the cervix, breast, oral cavity, lung, liver, colon, and rectum. Since 2008, the long‐form database has started to cover the prostate, esophagus, and bladder cancers. It broadened further to include cancers of the nasopharynx, salivary gland, uterus, ovary, and hematologic system in 2009. In this study, we utilized both short‐ and long‐form databases to extract the information on cancer diagnosis, staging, and treatment.

The National Birth Reporting Database (NBRD) of Taiwan was established in 1994. It includes information from all live‐ or still‐births with a gestational age of 20 weeks or older delivered in hospitals or clinics in Taiwan, obligatorily reported by doctors. The NBRD provides gender, birth weight, gestational age, newborn's medical parameters, and complications during pregnancy and delivery.

The Cause‐of‐Death Database (CODD) of Taiwan collects the time, location, and underlying cause of each induvial death, as reported in the death certificate. To protect the patient's privacy, information that can distinguish an individual's identity, such as name or address, was removed. An encrypted identification number can link the three datasets (TCR, NBRD, and CODD) to integrate information from the same individual. We performed the dataset linkage and data analysis in an access‐restricted room after the approval from the Institute Review Board of National Cheng Kung University Hospital (NCKUH‐A‐ER‐106‐289), and the Center for Health and Welfare Data Analysis and Application, Ministry of Health and Welfare, Taiwan.

### 2.2 Patient selection

We followed the eligibility criteria used in a previous study from Norway to identify the study cohort.^3^ First, we retrospectively identified women with cancer diagnosed at childbearing age (16–49 years) between 2001 and 2015 from TCR. Patients with non‐invasive cancer, patients with cancer diagnosed by image or autopsy, and patients with a history of previous cancer were excluded. After linkage with the NBRD, each patient in the study population was further categorized into three groups: (a) cancer diagnosed during pregnancy, (b) cancer diagnosed during the first postpartum year, and (c) cancer not associated with pregnancy. The duration of pregnancy was calculated by the date of delivery and the reported gestational week.

If a patient fulfilled the criteria for both group 1 and group 2 (ex. A woman diagnosed with cancer 11 months after the delivery of her first baby and was also pregnant at the time of cancer diagnosis), the patient would be categorized into the pregnancy group.

### 2.3 Survival and statistics analysis

We categorized the extent of disease into localized (stage 1 or 2), regional (stage 3), and metastatic (stage 4), and compared the extents of disease in different groups with ordinal logistic regression. The Mann‐Kendall method was used to test the time trend of incidence and average age during the study period. We compared each group's age with the one‐way analysis of variance (ANOVA) and categorical data with the chi‐squared test.

The overall survival was calculated by the difference between the date of cancer diagnosis and the time of death. Patients without death records in CODD were considered alive at the latest database update (31st December 2016). We used the Kaplan‐Meier method for survival analysis and compared the patients' survival in the three groups with the log‐rank test and the Cox proportional hazards model. Age, diagnostic year, and the initial extend of disease were adjusted in the multivariate analysis. The data manipulation and analysis were performed in software R version 3.5.1.

## 3 RESULTS

### 3.1 Incidence of PAC

**FIGURE 1:**
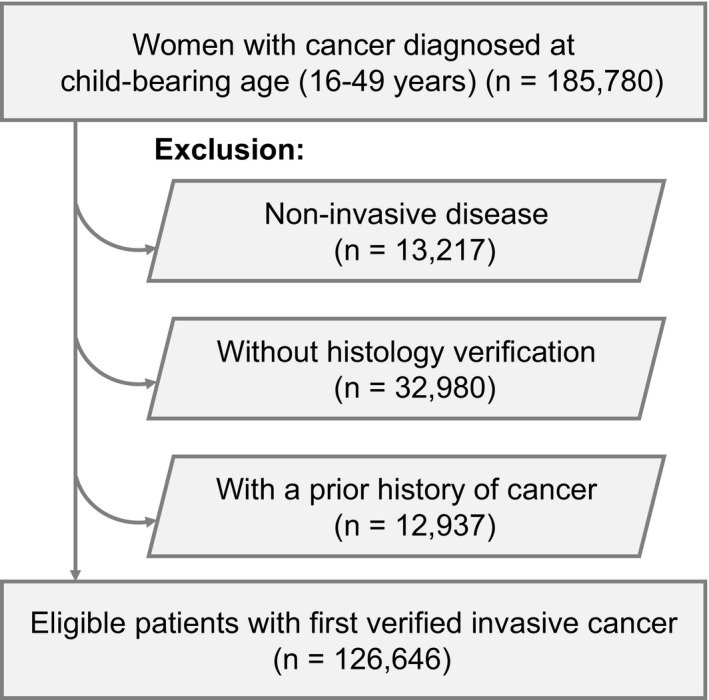
Flowchart of patient selection and exclusion

**FIGURE 2:**
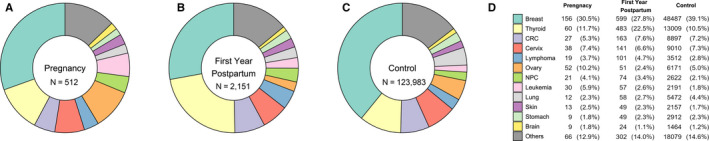
Distribution of cancer types in (A) pregnancy, (B) first year postpartum, and (C) control groups. (D) Summary of the number and percentage of each cancer type in each group

**FIGURE 3:**
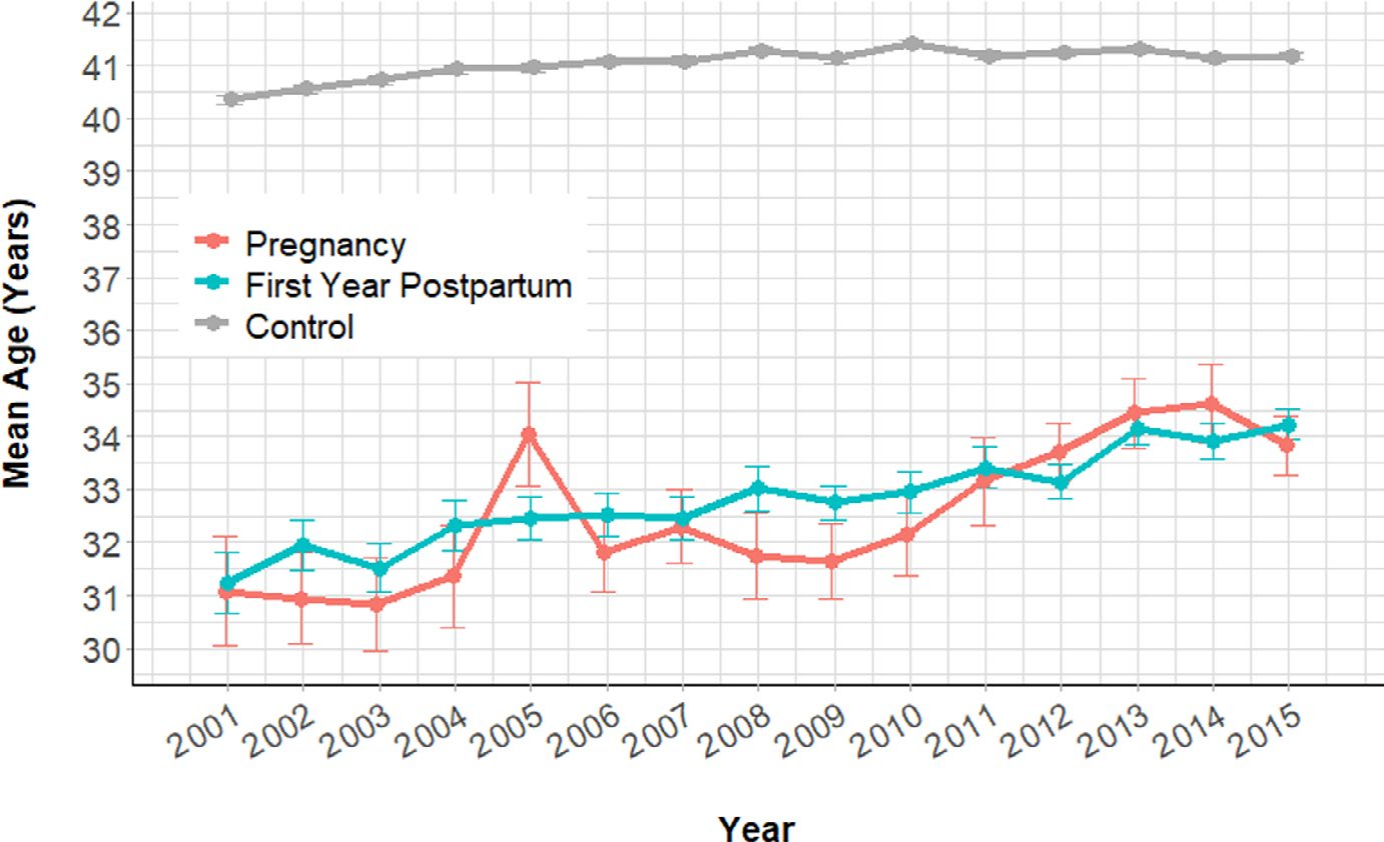
Mean age and standard error of the pregnancy, first year postpartum, and control groups

**FIGURE 4:**
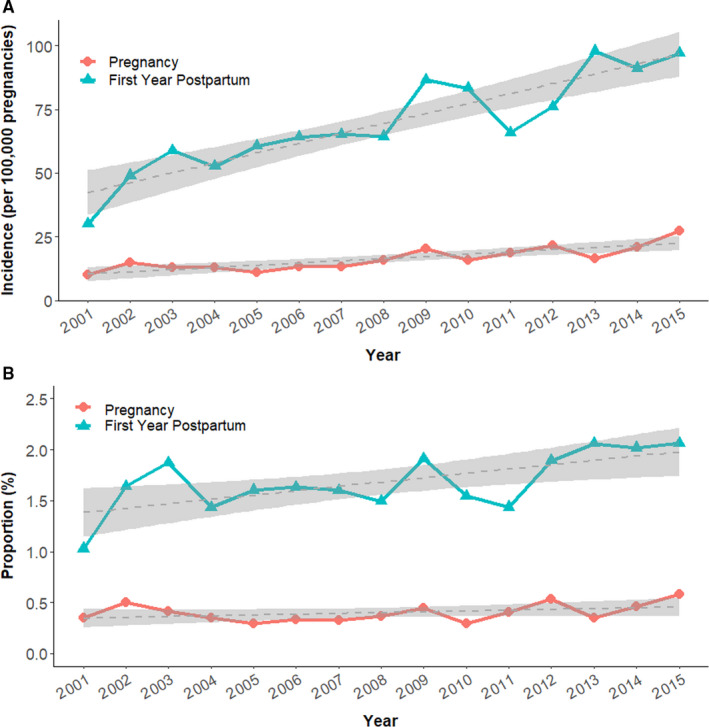
(A) Annual incidence of pregnancy‐associated cancers between 2001 and 2015. (B) The proportion of pregnancy‐associated cancers among all invasive malignancies diagnosed in women of childbearing age between 2001 and 2015

We identified female patients with the first invasive cancer diagnosed during childbearing age (16–49 years) between 2001 and 2015 (Figure 1). Among the 126,646 eligible patients, the malignancy was diagnosed during pregnancy in 512, and during the first postpartum year in 2151. The remaining 123,983 patients whose diseases were not pregnancy‐associated were regarded as the control group. Patients in the control group were older than patients in the pregnancy and postpartum group (Table 1) (*p* < 0.01, average 41.1 vs. 32.7 vs. 33.0 years, respectively). The number and proportion of different cancer types in each group are summarized in Figure 2A–D. Breast cancer was the most common cancer diagnosed during pregnancy and the first year postpartum (*N* = 156 and 599), followed by thyroid cancer (*N* = 60 and 483), colorectal cancer (CRC) (*N* = 27 and 163), cervical cancer (*N* = 38 and 141), and lymphoma (*N* = 19 and 101, respectively). During the study period, all three groups' mean age increased (Figure 3, *p* < 0.01 in all groups). The incidence of cancer diagnosed during pregnancy also elevated from 10.1 cases per 100,000 pregnancies in 2001 to 27.3 in 2015 (Figure 4A, *p* < 0.01). Similarly, the increasing trend existed in the postpartum group, with the incidence elevated from 30.0 to 97.0 cases per 100,000 pregnancies during 2001–2015 (Figure 4A, *p* < 0.01). The percentage of the postpartum group among the entire study population was also increasing, while the proportion of the pregnancy group was not (Figure 4B, *p* = 0.04 and 0.28, respectively). On average, the incidence of PAC throughout the study period was 84.8 cases per 100,000 pregnancies. In other words, about one in every 1200 pregnancies was complicated with cancer.

**TABLE 1:** Basic characteristics of patients in different groups

Group	Pregnancy (*n* = 512)	First year postpartum (*n* = 2151)	Control (*n* = 123,983)
Age (mean ± SD)*	32.7 ± 4.62	33.0 ± 4.67	41.1 ± 6.97
Age, median (range)	33 (18–46)	33 (17–49)	43 (16–49)
Age group, *n* (%)			
<30 years	125 (24)	475 (22)	9739 (8)
30–39 years	348 (68)	1517 (71)	31,047 (25)
≥40 years	39 (8)	159 (7)	83,197 (67)
Year of diagnosis, *n* (%)			
2001–2005	141 (28)	563 (26)	36,493 (29)
2006–2010	150 (29)	693 (32)	41,482 (34)
2011–2016	221 (43)	895 (42)	46,008 (37)
Trimester of diagnosis, *n* (%)			
Trimester 1 (<13 weeks)	76 (15)	NA	NA
Trimester 2 (13–26 weeks)	183 (36)	NA	NA
Trimester 3 (>26 weeks)	253 (49)	NA	NA

* *p* < 0.01.

### 3.2 The extent of disease at diagnosis

For all cancer types combined, patients in both pregnancy and first postpartum year groups had higher extents of disease than patients of the control group (odds ratio [OR] 1.35, 95% confidence interval [CI] 1.02–1.77, *p* = 0.03; OR 1.36, 95% CI 1.18–1.57, *p* < 0.01, respectively) (Table 2). As for each cancer, breast cancers diagnosed during antenatal or postpartum periods were more likely to be in advanced stages. Compared with 23.2% in the control group, 37.2% in the pregnancy group and 33.8% in the postpartum group were in advanced stages at diagnosis (OR 2.11, 95% CI 1.37–3.18, *p* < 0.01; OR 1.63, 95% CI 1.32–2.00, *p* < 0.01, respectively). The majority of CRC patients diagnosed during pregnancy were in advanced stages (15.4% regional and 76.9% metastatic), leads to an OR of 11.11 (95% CI 2.97–71.97, *p* < 0.01) compared with patients in the control group. A trend toward localized stages could be observed in patients with ovarian cancer diagnosed during pregnancy (OR 0.30, 95% CI 0.09–0.79, *p* = 0.03) and the postpartum period (OR 0.43, 95% CI 0.14–1.08, *p* = 0.10). There was no significant difference between each group in lymphoma and cancers of cervix, nasopharynx, and lung. Due to the small numbers of patients with stage data in each group, we could not perform meaningful comparison in the skin and gastric cancer (GC) subgroups.

**TABLE 2:** Initial extent of disease of patients in different groups

	*N*	Localized (%)	Regional (%)	Metastatic (%)	No data (%)	OR	95% CI	*p*‐value
All								
Control	63,263	49.5	15.8	11.7	23.0	1^a^		
Prengnancy	286	39.5	15.7	13.6	31.1	1.35	1.02–1.77	0.03
First year postpartum	1199	35.9	14.7	12.4	36.9	1.36	1.18–1.57	<0.01
Breast								
Control	25,582	71.5	17.6	5.6	5.3	1^a^		
Pregnancy	92	57.4	23.4	13.8	5.3	2.11	1.37–3.18	<0.01
First year postpartum	337	63.6	25.0	8.8	2.5	1.63	1.32–2.00	<0.01
Thyroid								
Control	7636	12.4	1.0	0.4	86.2	1^a^		
Pregnancy	33	13.2	0.0	0.0	86.8	NA	NA	NA
First year postpartum	289	11.8	0.0	0.0	88.2	NA	NA	NA
Colon & rectum								
Control	4609	23.8	38.7	29.1	8.4	1^a^		
Pregnancy	16	0.0	15.4	76.9	7.7	11.11	2.97–71.97	<0.01
First year postpartum	95	24.1	33.0	38.4	4.5	1.27	0.89–1.84	0.19
Cervix								
Control	3154	64.5	18.4	10.2	6.9	1^a^		
Pregnancy	21	60.9	13.0	4.3	21.7	0.63	0.18–1.73	0.41
First year postpartum	79	66.7	11.5	12.6	9.2	0.87	0.52–1.41	0.59
Lymphoma								
Control	1747	42.6	10.7	19.2	27.6	1^a^		
Pregnancy	8	44.4	22.2	11.1	22.2	0.87	0.18–3.38	0.85
First year postpartum	53	42.2	14.1	17.2	26.6	0.99	0.55–1.72	0.97
Ovary								
Control	3220	45.6	22.2	7.1	25.1	1^a^		
Pregnancy	35	60	8.6	2.9	28.6	0.30	0.09–0.79	0.03
First year postpartum	27	57.6	9.1	6.1	27.3	0.43	0.14–1.08	0.10
Nasopharynx								
Control	1121	21.3	26.8	24.9	27.0	1^a^		
Pregnancy	10	10.0	40.0	20.0	30.0	1.19	0.33–4.49	0.79
First year postpartum	38	8.0	32.0	26.0	34.0	1.60	0.87–3.00	0.13
Lung								
Control	2927	26.8	12.5	58.1	2.7	1^a^		
Pregnancy	5	16.7	0.0	83.3	0.0	3.11	0.50–59.85	0.30
First year postpartum	36	35.9	5.1	48.7	10.3	0.7	0.36–1.37	0.28
Skin								
Control	994	6.3	0.4	0.4	93.0	1^a^		
Pregnancy	5	0.0	0.0	0.0	100.0	NA	NA	NA
First year postpartum	20	0.0	0.0	0.0	100.0	NA	NA	NA
Stomach								
Control	1227	25.4	16.8	36.8	20.9	1^a^		
Pregnancy	3	0.0	0.0	60.0	40.0	NA	NA	NA
First year postpartum	22	4.3	21.7	56.5	17.4	NA	NA	NA
Others								
Control	9979	37.1	8.8	9.2	44.9	1^a^		
Pregnancy	41	19.0	11.9	9.5	59.5	2.02	0.82–4.78	0.11
First year postpartum	165	21.1	8.0	9.0	61.8	1.64	1.05–2.52	0.03

The table was generated with patients diagnosed between 2009 and 2015 because of data integrity. There were no data of stage in leukemia and brain cancers. NA was generated due to low case numbers.

Abbreviations: CI, confidence interval; NA, not available; OR, odds ratio.

^a^ Reference group.

### 3.3 Overall survival of PAC

**FIGURE 5:**
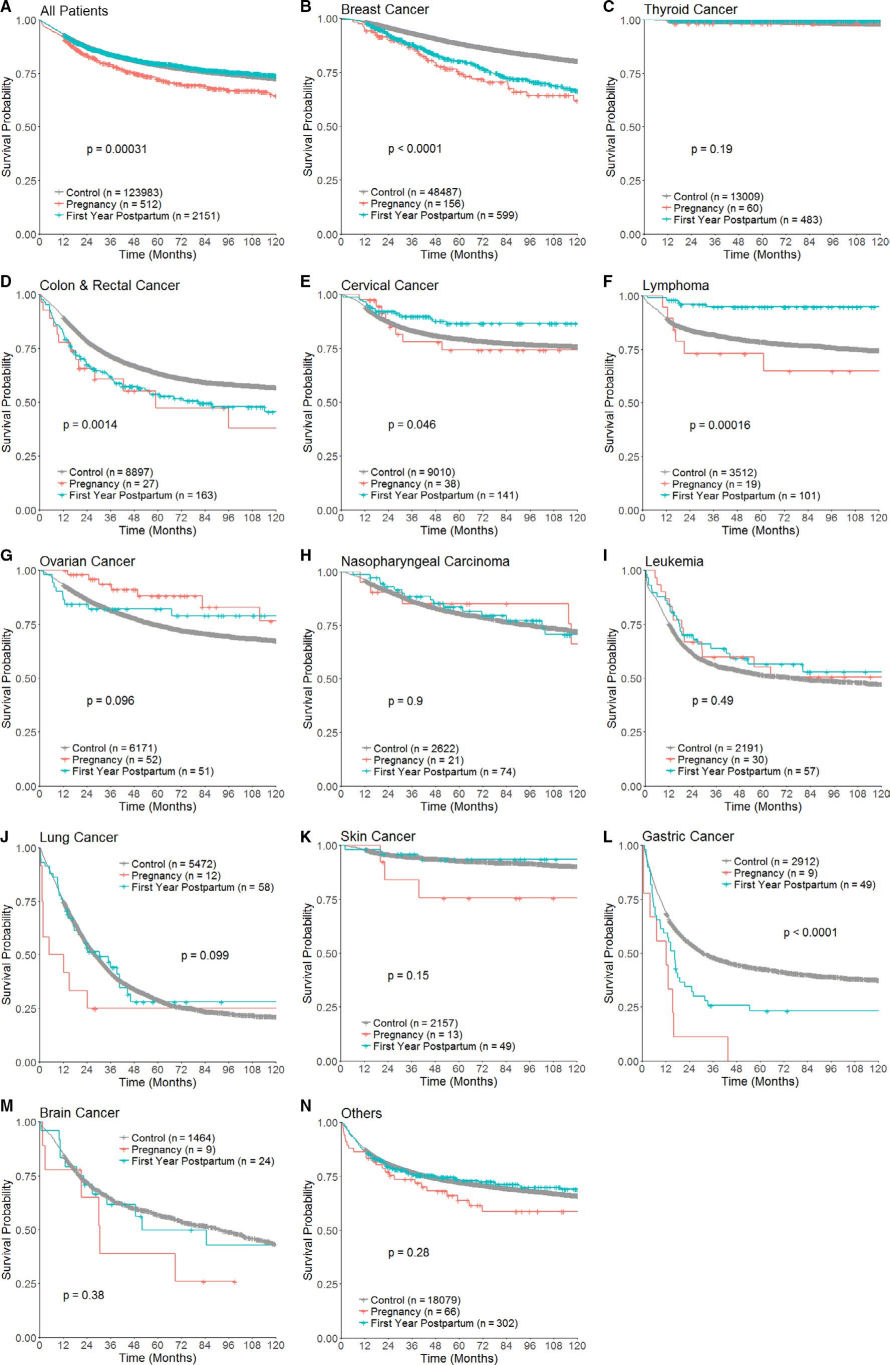
Kaplan‐Meier survival curves of patients of each cancer type in the pregnancy, first year postpartum, and control groups. Log‐rank test *p* values are displayed

With all cancer types combined and the control group as a reference, a worse overall survival can be observed in patients with cancer diagnosed during pregnancy (Hazard ratio [HR] 1.37, 95% CI 1.17–1.62, *p* < 0.01), but not in patients of the postpartum group (HR 0.97, 95% CI 0.88–1.06, *p* = 0.45). The hazard ratios turned insignificant after adjustment with age, stage, and the year of diagnosis (Table 3; Figure 5A).

**TABLE 3:** Overall survival of patients in different groups

	*N*	Crude HR	95% CI	*p*‐value^a^	*p*‐value^b^	Adjusted HR	95% CI	*p*‐value^a^
All								
Control	123,983	1^c^				1^c^		
Pregnancy	512	1.37	1.17–1.62	<0.01		1.07	0.80–1.41	0.65
First year postpartum	2151	0.97	0.88–1.06	0.45	<0.01	1.02	0.88–1.18	0.77
Breast								
Control	48,487	1^c^				1^c^		
Pregnancy	156	2.31	1.71–3.12	<0.01		1.02	0.64–1.62	0.95
First year postpartum	599	1.88	1.60–2.21	<0.01	<0.01	1.34	1.05–1.72	0.02
Thyroid								
Control	13,009	1^c^				1^c^		
Pregnancy	60	1.08	0.15–7.74	0.94		1.81	0.25–13.09	0.56
First year postpartum	483	0.30	0.07–1.19	0.09	0.19	0.46	0.11–1.86	0.27
CRC								
Control	8897	1^c^				1^c^		
Pregnancy	27	1.67	0.97–2.88	0.07		0.80	0.38–1.68	0.55
First year postpartum	163	1.44	1.14–1.80	<0.01	<0.01	1.08	0.80–1.46	0.60
Cervix								
Control	9010	1^c^				1^c^		
Pregnancy	38	1.16	0.60–2.23	0.66		2.09	0.86–5.07	0.10
First year postpartum	141	0.56	0.35–0.90	0.02	0.05	0.40	0.20–0.82	0.01
Lymphoma								
Control	3528	1^c^				1^c^		
Pregnancy	19	1.41	0.63–3.15	0.40		1.51	0.21–10.88	0.68
First year postpartum	101	0.19	0.08–0.47	<0.01	<0.01	0.11	0.02–0.79	0.03
Ovary								
Control	6171	1^c^				1^c^		
Pregnancy	52	0.48	0.23–1.01	0.05		0.73	0.18–2.95	0.66
First year postpartum	51	0.75	0.40–1.40	0.37	0.10	1.04	0.33–3.25	0.95
Nasopharynx								
Control	2622	1^c^				1^c^		
Pregnancy	21	1.15	0.51–2.56	0.74		0.97	0.13–7.00	0.97
First year postpartum	74	0.92	0.56–1.51	0.75	0.90	1.05	0.42–2.60	0.92
Leukemia								
Control	2,191	1^c^				1^c^		
Pregnancy	30	0.88	0.52–1.48	0.62		0.99	0.58–1.68	0.96
First year postpartum	57	0.86	0.57–1.28	0.45	0.49	0.91	0.60–1.36	0.64
Lung								
Control	5472	1^c^				1^c^		
Pregnancy	12	2.02	1.05–3.89	0.04		2.71	1.01–7.28	0.05
First year postpartum	58	1.01	0.73–1.41	0.93	0.09	1.25	0.79–1.98	0.34
Skin								
Control	2157	1^c^				1^c^		
Pregnancy	13	2.84	0.91–8.89	0.07		3.77	1.18–12.02	0.02
First year postpartum	49	0.73	0.23–2.28	0.59	0.15	0.93	0.29–2.97	0.91
Stomach								
Control	2912	1^c^				1^c^		
Pregnancy	9	3.15	1.64–6.07	<0.01		2.23	0.70–7.08	0.17
First year postpartum	49	1.59	1.15–2.21	<0.01	<0.01	1.48	0.91–2.43	0.12
Primary brain								
Control	1464	1^c^				1^c^		
Pregnancy	9	1.76	0.79–3.93	0.17		1.93	0.86–4.32	0.11
First year postpartum	24	0.99	0.56–1.75	0.97	0.38	1.03	0.58–1.83	0.92
Others								
Control	18,063	1^c^				1^c^		
Pregnancy	66	1.34	0.91–1.99	0.14		1.21	0.60–2.44	0.59
First year postpartum	302	0.94	0.76–1.17	0.57	0.29	1.24	0.82–1.88	0.31

Univariate analyses are presented as crude HR at the left column. Multivariate analyses are adjusted for age, diagnostic year, and extend of disease at diagnosis (presented at the right column).

Abbreviations: CI, confidence interval; HR, hazard ratio.

^a^ Cox proportional hazards.

^b^ Log‐rank test.

^c^ Reference group.

As for each site of cancer diagnosed during pregnancy, we observed an elevated risk of death in patients of breast cancer (Figure 5B; HR 2.31, 95% CI 1.71–3.12, *p* < 0.01), lung cancer (Figure 5J; HR 2.02, 95% CI 1.05–3.89, *p* = 0.04), and gastric cancer (Figure 5L; HR 3.15, 95% CI 1.64–6.07, *p* < 0.01). Similarly, an increased risk of death could also be seen in patients with breast cancer (Figure 5B; HR 1.88, 95% CI 1.60–2.21, *p* < 0.01), CRC (Figure 5D; HR 1.44, 95% CI 1.14–1.80, *p* < 0.01), and gastric cancer (Figure 5L; HR 1.59, 95% CI 1.15–2.21, *p* < 0.01) diagnosed during the first postpartum year. After adjustment with age, stage, and diagnostic year, the inferior outcome remained significant in patients with skin cancer diagnosed during pregnancy (adjusted HR 3.77, 95% CI 1.18–12.02, *p* = 0.02) and postpartum breast cancer (Table 3; adjusted HR 1.34, 95% CI 1.05–1.72, *p* = 0.02).

In contrast, patients with postpartum cervical cancer (Figure 5E; HR 0.56, 95% CI 0.35–0.90, *p* = 0.02) and lymphoma (Figure 5F; HR 0.19 95% CI 0.08–0.47, *p* < 0.01) had better outcomes than patients without pregnancy. The differences were significant in the multivariate analysis, with adjusted HR 0.40 (95% CI 0.20–0.82, *p* = 0.01) for cervical cancer, and HR 0.11 (95% CI 0.02–0.79, *p* = 0.03) for lymphoma (Table 3).

**FIGURE 6:**
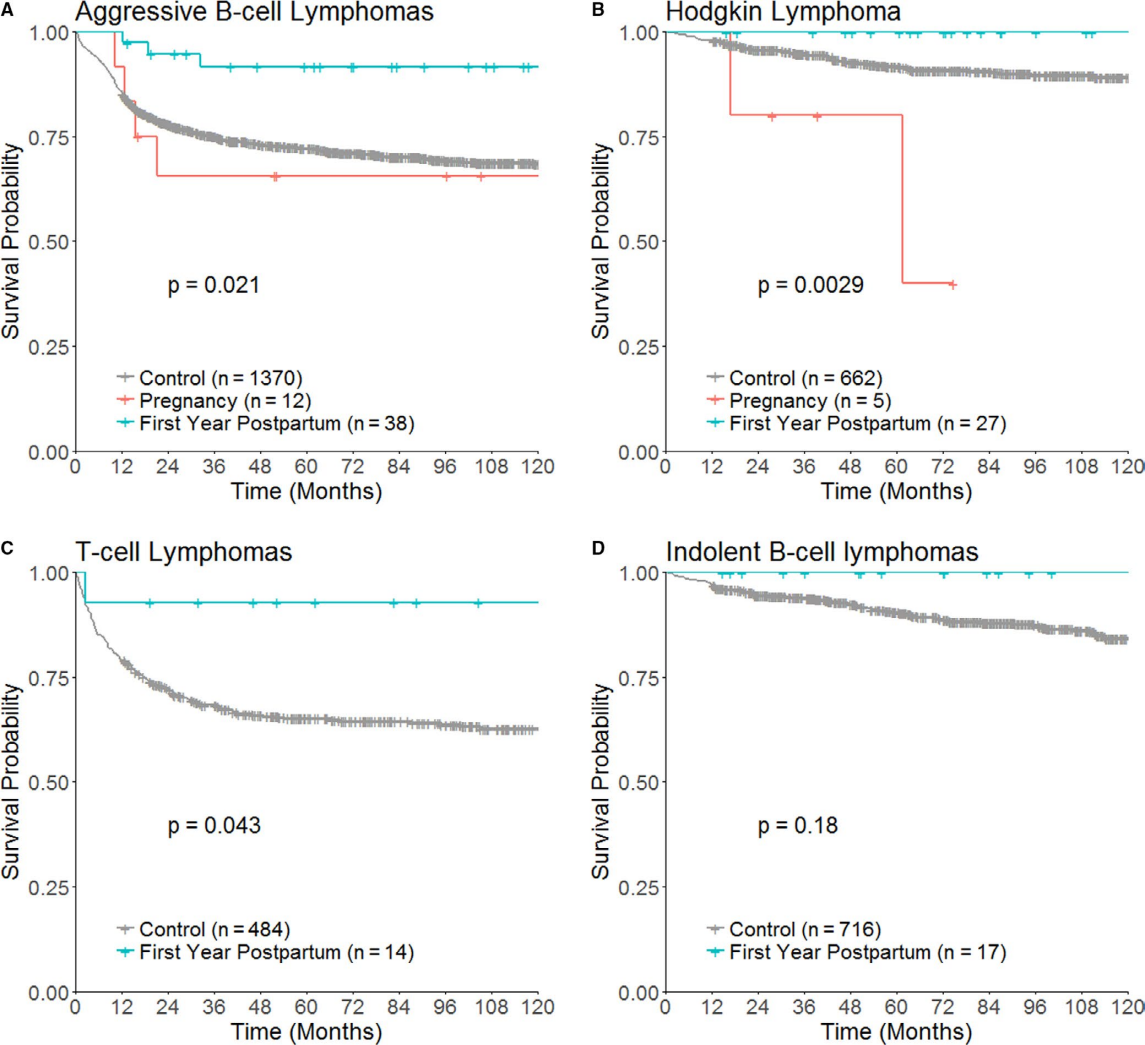
Kaplan‐Meier survival curves of patients of different subtypes of lymphoma in the pregnancy, first year postpartum, and control groups. Log‐rank test *p* values are displayed

The trend toward better survival could be observed in patients aggressive B‐cell lymphomas (Figure 6A; HR 0.23, 95% CI 0.07–0.72, *p* = 0.01) and T‐cell lymphomas (Figure 6C; HR 0.17, 95% CI 0.02–1.20, *p* = 0.07), but not in Hodgkin lymphoma (Figure 6B; HR < 0.01, 95% CI 0‐Infinity, *p* = 0.99) and indolent lymphomas (Figure 6D; HR < 0.01, 95% CI 0‐infinity, *p* = 0.994). The survival of patients with leukemia, thyroid, ovarian, nasopharyngeal, primary brain, and other cancers is not affected by pregnancy or postpartum status (Figure 5C,G,H,I,M,N). Analysis performed in the subgroup of 2009–2015 showed comparable results as the study cohort (Table S1).

## 4 DISCUSSION

In this study, we identified a total of 2663 patients with PAC, and the overall crude incidence was 84.8 cases per 100,000 pregnancies, which was lower than most previous studies that generally reported an incidence above 100.^3^, ^34^, ^35^ The difference might be primarily due to the ethnic difference in the incidence of malignant melanoma between Caucasian and Asian populations. In previous reports, malignant melanoma is common and is even the most common PAC in Scandinavian countries and Australia.^3^, ^7^, ^34^ In contrast, the most common cancer diagnosed during pregnancy in the Taiwan population was breast cancer, while pregnancy‐associated melanoma was rare. Our result was different from the studies of other East Asian countries. The most common cancer diagnosed during pregnancy is breast cancer in a single‐institute report from Korea,^31^ is leukemia in a multi‐center study from China,^32^ and is cervical cancer in a multi‐center survey from Japan.^30^ However, contrary to the nationwide population‐based cohort used in our study, all these reports are limited with small case numbers.

During the study period, PAC's overall incidence increased, from 40.1 cases per 100,000 pregnancies in 2001 to 124.3 in 2015. The increasing incidence of PAC was also observed in the reports from Norway, Australia, and the United States.^3^, ^5^, ^34^ We believe it is associated with the trend of delay childbearing and the elevating incidence of cancer.^36^, ^37^ The average age of childbearing women in Taiwan has increased from 28.2 in 2001 to 32.0 years old in 2018, as well as the age at first pregnancy (elevated from 26.9 to 30.9).^38^ We showed an increasing average age of PAC patients in our study, and there's also a rising proportion of postpartum cancer among all cancer in women of childbearing age. Therefore, the incidence of PAC is expected to increase in the future and warrant more attention.

Breast cancer is the most common PAC in Taiwan, and pregnancy‐associated breast cancer is more likely to be in advanced stages and have poorer outcomes. During pregnancy and lactation, a breast lump or inflammation may be regarded as normal changes rather than malignancy, and patients with pregnancy‐associated breast cancer may experience diagnostic delays.^39^ The initiation of breast cancer treatment in pregnant women is also likely to be delayed.^40^ Beyond delay in diagnosis and treatment, the higher risk of metastasis and death in young women's breast cancer,^41^, ^42^, ^43^ and the higher proportion of hormone negative and HER2‐positive subtypes also contribute to the worse outcome of pregnancy‐associated breast cancer.^13^

In our study, patients with breast cancer diagnosed during pregnancy had similar survival with patients without pregnancy after controlling stage, age, and the year of diagnosis. Conversely, the diagnosis of breast cancer during the first postpartum year was an independent poor prognostic factor. The result was consistent with previous studies and highlighted the importance of judging these two groups differently.^3^, ^13^, ^26^, ^27^ Patients diagnosed postpartum have a higher risk of recurrence and metastasis,^26^ with a distinct metastatic preference to the liver.^44^ At the end of milk production, the mammary gland undergoes a tissue remodeling process known as involution.^45^ Evidence from preclinical studies suggests that the microenvironment during involution promotes tumorigenesis and increases the risk of metastasis.^44^, ^46^, ^47^, ^48^ The underlying molecular mechanism warrants further exploration to discover a way to prevent or treat postpartum breast cancer in the future.^49^

Consistent with previous reports, patients with pregnancy‐associated GC and CRC in our cohort have poor survival.^50^, ^51^ More than 70% of patients with pregnancy‐associated CRC or GC were in an advanced stage at diagnosis in our cohort, suggesting a delay in diagnosis. During pregnancy, symptoms of gastrointestinal cancers, such as vomiting, constipation, and rectal bleeding, are easily overlooked and attributed to pregnancy. Early diagnosis of CRC or GC in pregnant women is challenging, while radiologic and endoscopic examinations may also be limited or delayed.

Although some early studies suggested a possible link between hormones and the aggressiveness of CRC,^52^, ^53^ recent evidence indicates the protective role of estrogen against the CRC carcinogenesis through estrogen receptor β (ER β) signaling.^54^, ^55^, ^56^ Moreover, a recent study reported a negative impact on survival with estrogen receptor α (ER α) expression in CRC, but not with progesterone receptor.^57^ In GC, the expression of ER α is also an indicator of poor prognosis.^58^, ^59^ These findings suggested a potential role of hormone exposure in pregnancy‐associated gastrointestinal cancers and warrant further investigation.

Cervical cancer is the fourth most common PAC in Taiwan. Like previous studies, our patients with cervical cancer diagnosed during pregnancy had a similar distribution of stages and survival with patients without pregnancy.^60^, ^61^, ^62^, ^63^ Interestingly, in our data, patients with postpartum cervical cancer had a significantly better outcome than the control group. The finding is contrary to two previous reports, one reported similar, and the other reported worse outcomes in the postpartum group.^3^, ^61^ The difference in the Pap smear rate between groups may be a possible explanation. The overall Pap smear rate in Taiwan is only around 50%,^64^ which confers to a higher incidence of cervical cancer in Taiwan than in developed countries.^65^ In our data, there is also a higher proportion of patients in advanced stages of cervical cancer than the report from Norway.^3^ On the contrary, women at perinatal care may have more frequent Obstetrics clinic visits and a higher likelihood to receive Pap smear, which may confer to a better outcome in such a group.

In our cohort, lymphoma patients who were diagnosed during pregnancy had similar survival with the control group. The result was consistent with previous reports on maternal outcomes after Hodgkin and non‐Hodgkin lymphomas (NHL).^66^, ^67^ Surprisingly, our results showed that patients with postpartum lymphoma had significantly better survival than the control group. The superiority was observed across different lymphoma subtypes and remained true after controlled with age and the extent of disease. The hormone changes during pregnancy and postpartum lactating period provide a possible explanation to the survival superiority in these patients. We could find clues in epidemiology studies showing that women have a lower incidence and better survival in most subtypes of lymphoma.^68^ Women taking oral contraceptives have an even lower risk of developing NHL than those who did not.^69^ Moreover, multi‐parity and early age at first birth are protective factors against NHL.^70^ In recent years, there is emerging evidence about the role of estrogen in B‐cell lymphomas.^71^, ^72^ The study from Yakimchuk et al. also provided in vitro evidence to suggest that targeting ER β with agonists may be a potential treatment for lymphoma.^73^

Our data showed similar survival and a trend toward the early stage for women with ovarian cancer diagnosed during pregnancy. The high proportion of localized disease echos the findings from other reports.^3^, ^74^, ^75^ During pregnancy, routine physical and ultrasound examinations provide a chance of early diagnosis of ovarian tumors. An ovarian tumor may also be noticed incidentally during the Caesarean section. In addition to early diagnosis, ovarian malignancies in young women have a predominance of germ cell and sex cord‐stromal tumors, which have a good prognosis.^76^. We did not see the worse prognosis in postpartum ovarian cancer patients, as shown in the report from Stensheim et al.^3^ However, the number of postpartum subgroups in both studies have small numbers, and the results should be interpreted with caution.

Our study had some limitations. First, the NBRD only report deliveries with a gestational age of more than 20 weeks. Patients who terminated gestation early may not be registered and lead to an under‐estimation of the PAC incidence, especially PAC diagnosed during the first trimester. However, the limit exists in most reports utilizing a similar approach, and the comparison between our data and other studies are reasonable. Second, the stage data is not available for all types of cancers, particularly in patients diagnosed before 2009. The limitation may affect the result of survival analysis after control with age, diagnosis period, and extent of the stage. Therefore, we performed a subgroup analysis in patients diagnosed between 2009 and 2015 (Table S1). The subgroup analysis did not change the trend and result obtained in most cancers.

Despite these limitations, our study provided the first population‐based evaluation of PAC from an Asian country. In conclusion, the incidence of PAC in Taiwan was increasing, with breast cancer being the most common. In general, the cancer diagnosis during pregnancy or the first postpartum year does not affect patients' survival with most cancer types. Exceptions include the worse prognosis of postpartum breast cancer and the better outcome of postpartum lymphoma and cervical cancer.

## CONFLICT OF INTEREST

The authors declare that they have no conflict of interest.

## Supporting information

Supplementary Material

## Data Availability

The data that support the findings of this study are available from the Center for Health and Welfare Data Analysis and Application, Ministry of Health and Welfare, Taiwan, but restrictions apply to the availability of these data, which were used under license for the current study, and so are not publicly available.

## References

[1] Parazzini F , Franchi M , Tavani A , Negri E , Peccatori FA . Frequency of pregnancy related cancer. Int J Gynecol Cancer. 2017;27(3):613–619. 28107260 10.1097/IGC.0000000000000904

[2] Botha MH , Rajaram S , Karunaratne K . Cancer in pregnancy. Int J Gynecol Obstet. 2018;143:137–142. 10.1002/ijgo.12621 30306590

[3] Stensheim H , Møller B , Van Dijk T , Fosså SD . Cause‐specific survival for women diagnosed with cancer during pregnancy or lactation: a registry‐based cohort study. J Clin Oncol. 2009;27(1):45–51. 19029418 10.1200/JCO.2008.17.4110

[4] Pavlidis NA . Coexistence of pregnancy and malignancy. Oncologist. 2002;7(4):279–287. 12185292

[5] Cottreau CM , Dashevsky I , Andrade SE , et al. Pregnancy‐associated cancer: a U.S. population‐based study. J Womens Health. 2019;28(2):250–257. 10.1089/jwh.2018.6962 PMC6390809 30307780

[6] Van Calsteren K , Heyns L , De Smet F , et al. Cancer during pregnancy: an analysis of 215 patients emphasizing the obstetrical and the Neonatal outcomes. J Clin Oncol. 2010;28(4):683–689. 19841323 10.1200/JCO.2009.23.2801

[7] Lu D , Ludvigsson JF , Smedby KE , et al. Maternal cancer during pregnancy and risks of stillbirth and infant mortality. J Clin Oncol. 2017;35(14):1522–1529. 28384079 10.1200/JCO.2016.69.9439

[8] Wu TP , Liang FW , Huang YL , Chen LH , Lu TH . Maternal mortality in Taiwan: a nationwide data linkage study. PLoS One. 2015;10(8):1–10. 10.1371/journal.pone.0132547 PMC4523206 26237411

[9] Morice P , Uzan C , Uzan S . Cancer in pregnancy: a challenging conflict of interest. Lancet. 2012;379(9815):495–496. 22325648 10.1016/S0140-6736(11)61814-X

[10] Moran BJ , Yano H , Al Zahir N , Farquharson M . Conflicting priorities in surgical intervention for cancer in pregnancy. Lancet Oncol. 2007;8(6):536–544. 17540305 10.1016/S1470-2045(07)70171-7

[11] Loibl S , Schmidt A , Gentilini O , et al. Breast cancer diagnosed during pregnancy adapting recent advances in breast cancer care for pregnant patients. JAMA Oncol. 2015;1(8):1145–1153. 26247818 10.1001/jamaoncol.2015.2413

[12] Iqbal J , Amir E , Rochon PA , Giannakeas V , Sun P , Narod SA . Association of the timing of pregnancy with survival in women with breast cancer. JAMA Oncol. 2017;3(5):659–665. 28278319 10.1001/jamaoncol.2017.0248 PMC5824205

[13] Chuang SC , Lin CH , Lu YS , Hsiung CA . Association of pregnancy and mortality in women diagnosed with breast cancer: a Nationwide Population Based Study in Taiwan. Int J Cancer. 2018;143(10):2416–2424. 30070358 10.1002/ijc.31777

[14] Weetman AP . The immunology of pregnancy. Thyroid. 1999;9(7):643–646. 10447007 10.1089/thy.1999.9.643

[15] Genin AS , Antoine M , Aractingi S , Rouzier R . Pregnancy stimulates tumor angiogenesis in breast carcinoma. Anticancer Res. 2014;34(1):125–131. 24403452

[16] Zygmunt M , Herr F , Münstedt K , Lang U , Liang OD . Angiogenesis and vasculogenesis in pregnancy. Eur J Obstet Gynecol Reprod Biol. 2003;110:S10–S18. 12965086 10.1016/s0301-2115(03)00168-4

[17] Mor G , Cardenas I . The immune system in pregnancy. Am J Reprod Immunol. 2011;63(6):425–433. 10.1111/j.1600-0897.2010.00836.x PMC3025805 20367629

[18] van Hasselt J , van Calsteren K , Heyns L , et al. Optimizing anticancer drug treatment in pregnant cancer patients: pharmacokinetic analysis of gestation‐induced changes for doxorubicin, epirubicin, docetaxel and paclitaxel. Ann Oncol. 2014;25(10):2059–2065. 24713311 10.1093/annonc/mdu140

[19] Genin A‐S , Lesieur B , Gligorov J , Antoine M , Selleret L , Rouzier R . Pregnancy‐associated breast cancers: do they differ from other breast cancers in young women? Breast Edinb Scotl. 2012;21(4):550–555. 10.1016/j.breast.2012.05.002 22698618

[20] Murphy CG , Mallam D , Stein S , et al. Current or recent pregnancy is associated with adverse pathologic features but not impaired survival in early breast cancer. Cancer. 2012;118(13):3254–3259. 22086863 10.1002/cncr.26654

[21] Kroman N , Mouridsen HT . Prognostic influence of pregnancy before, around, and after diagnosis of breast cancer. Breast Edinb Scotl. 2003;12(6):516–521. 10.1016/s0960-9776(03)00159-0 14659129

[22] Callihan EB , Gao D , Jindal S , et al. Postpartum diagnosis demonstrates a high risk for metastasis and merits an expanded definition of pregnancy‐associated breast cancer. Breast Cancer Res Treat. 2013;138(2):549–559. 23430224 10.1007/s10549-013-2437-x PMC3608871

[23] Beadle BM , Woodward WA , Middleton LP , et al. The impact of pregnancy on breast cancer outcomes in women ≤35 years. Cancer. 2009;115(6):1174–1184. 19204903 10.1002/cncr.24165 PMC3063387

[24] Costa MM , Saldanha P . Breast cancer in pregnancy. Breast Dis Manag Ther. 2016;379(9815):405–414.

[25] Al Hadidi S . Timing of pregnancy and survival in women with breast cancer. JAMA Oncol. 2018;4(1):131. 10.1001/jamaoncol.2017.2711 28880981

[26] Amant F , von Minckwitz G , Han SN , et al. Prognosis of women with primary breast cancer diagnosed during pregnancy: results from an international collaborative study. J Clin Oncol Off J Am Soc Clin Oncol. 2013;31(20):2532–2539. 10.1200/JCO.2012.45.6335 23610117

[27] Azim HA , Santoro L , Russell‐Edu W , Pentheroudakis G , Pavlidis N , Peccatori FA . Prognosis of pregnancy‐associated breast cancer: a meta‐analysis of 30 studies. Cancer Treat Rev. 2012;38(7):834–842. 22785217 10.1016/j.ctrv.2012.06.004

[28] Yap Y‐S , Lu Y‐S , Tamura K , et al. Insights into breast cancer in the east vs the west: a review. JAMA Oncol. 2019;5(10):1489. 10.1001/jamaoncol.2019.0620 31095268

[29] Huh J . Epidemiologic overview of malignant lymphoma. Korean J Hematol. 2012;47(2):92. 22783355 10.5045/kjh.2012.47.2.92 PMC3389073

[30] Kobayashi Y , Tabata T , Omori M , et al. A Japanese survey of malignant disease in pregnancy. Int J Clin Oncol. 2019;24(3):328–333. 30368627 10.1007/s10147-018-1352-x

[31] Shim MH , Mok C‐W , Chang K‐J , et al. Clinical characteristics and outcome of cancer diagnosed during pregnancy. Obstet Gynecol Sci. 2016;59(1):1. 26866029 10.5468/ogs.2016.59.1.1 PMC4742470

[32] Yp Z , J D , Xw Z , J LI , Y S . Maternal and neonatal outcomes of cancer during pregnancy: a multi‐center observational study. J Cancer. 2019;10(23):5727–5734. 31737109 10.7150/jca.33746 PMC6843891

[33] Chiang CJ , Wang YW , Lee WC . Taiwan's Nationwide Cancer Registry System of 40 years: past, present, and future. J Formos Med Assoc. 2019;118(5):856–858. 30773272 10.1016/j.jfma.2019.01.012

[34] Lee YY , Roberts CL , Dobbins T , et al. Incidence and outcomes of pregnancy‐associated cancer in Australia, 1994–2008: a population‐based linkage study. BJOG Int J Obstet Gynaecol. 2012;119(13):1572–1582. 10.1111/j.1471-0528.2012.03475.x PMC3533794 22947229

[35] Smith LH , Danielsen B , Allen ME , Cress R . Cancer associated with obstetric delivery: results of linkage with the California cancer registry. Am J Obstet Gynecol. 2003;189(4):1128–1135. 14586366 10.1067/s0002-9378(03)00537-4

[36] Liu F‐C , Lin H‐T , Kuo C‐F , See L‐C , Chiou M‐J , Yu H‐P . Epidemiology and survival outcome of breast cancer in a nationwide study. Oncotarget. 2017;8(10):16939–16950. 28199975 10.18632/oncotarget.15207 PMC5370012

[37] Chiang C‐J , Lo W‐C , Yang Y‐W , You S‐L , Chen C‐J , Lai M‐S . Incidence and survival of adult cancer patients in Taiwan, 2002–2012. J Formos Med Assoc. 2016;115(12):1076–1088. 26786251 10.1016/j.jfma.2015.10.011

[38] Statistics of the Department of Household Registration, Ministry of Interior, Taiwan [Internet]. [cited 2020 Mar 1]. https://www.ris.gov.tw/app/en/3911

[39] Amant F , Loibl S , Neven P , Calsteren KV . Breast cancer in pregnancy. Lancet. 2012;379(9815):570–579. 22325662 10.1016/S0140-6736(11)61092-1

[40] Yang YL , Chan KA , Hsieh FJ , Chang LY , Wang MY . Pregnancy‐associated breast cancer in Taiwanese women: Potential treatment delay and impact on survival. PLoS One. 2014;9(11):1–13. 10.1371/journal.pone.0111934 PMC4240543 25415309

[41] Anders CK , Hsu DS , Broadwater G , et al. Young age at diagnosis correlates with worse prognosis and defines a subset of breast cancers with shared patterns of gene expression. J Clin Oncol. 2008;26(20):3324–3330. 18612148 10.1200/JCO.2007.14.2471

[42] Azim HA , Michiels S , Bedard PL , et al. Elucidating prognosis and biology of breast cancer arising in young women using gene expression profiling. Clin Cancer Res. 2012;18(5):1341–1351. 22261811 10.1158/1078-0432.CCR-11-2599

[43] Bharat A , Aft RL , Gao F , Margenthaler JA . Patient and tumor characteristics associated with increased mortality in young women (≤40 years) with breast cancer: young women with breast cancer. J Surg Oncol. 2009;100(3):248–251. 19330813 10.1002/jso.21268

[44] Goddard ET , Hill RC , Nemkov T , et al. The rodent liver undergoes weaning‐induced involution and supports breast cancer metastasis. Cancer Discov. 2017;7(2):177–187. 27974414 10.1158/2159-8290.CD-16-0822 PMC5459606

[45] Watson CJ . Key stages in mammary gland development – involution: apoptosis and tissue remodelling that convert the mammary gland from milk factory to a quiescent organ. Breast Cancer Res. 2006;8(2):203. 16677411 10.1186/bcr1401 PMC1557708

[46] Schedin P . Pregnancy‐associated breast cancer and metastasis. Nat Rev Cancer. 2006;6(4):281–291. 16557280 10.1038/nrc1839

[47] Lyons TR , O'Brien J , Borges VF , et al. Postpartum mammary gland involution drives progression of ductal carcinoma in situ through collagen and COX‐2. Nat Med. 2011;17(9):1109–1115. 21822285 10.1038/nm.2416 PMC3888478

[48] Lyons TR , Borges VF , Betts CB , et al. Cyclooxygenase‐2–dependent lymphangiogenesis promotes nodal metastasis of postpartum breast cancer. J Clin Invest. 2014;124(9):3901–3912. 25133426 10.1172/JCI73777 PMC4153700

[49] Borges VF , Lyons TR , Germain D , Schedin P . Postpartum involution and cancer: an opportunity for targeted breast cancer prevention and treatments? Cancer Res. 2020;80(9):1790–1798. 32075799 10.1158/0008-5472.CAN-19-3448 PMC8285071

[50] Pellino G , Simillis C , Kontovounisios C , et al. Colorectal cancer diagnosed during pregnancy: systematic review and treatment pathways. Eur J Gastroenterol Hepatol. 2017;29(7):743–753. 28252463 10.1097/MEG.0000000000000863

[51] Gabriel I , Olejek A , Drozdzowska B . Colon cancer in pregnancy – a difficult diagnosis. Eur J Obstet Gynecol Reprod Biol. 2016;203:340–341. 10.1016/j.ejogrb.2016.05.051 27349170

[52] Dawson PM , Shousha S , Blair SD , et al. Oestrogen receptors in colorectal carcinoma. J Clin Pathol. 1990;43(2):149–151. 2180984 10.1136/jcp.43.2.149 PMC502298

[53] Slattery ML , Samowitz WS , Holden JA . Estrogen and progesterone receptors in colon tumors. Am J Clin Pathol. 2000;113(3):364–368. 10705816 10.1309/5MHB-K6XX-QV50-PCJQ

[54] Hartman J , Gustafsson JÅ . Estrogen receptors in colorectal cancer: goalkeepers, strikers, or bystanders? Cancer Prev Res. 2010;3(8):897–899. 10.1158/1940-6207.CAPR-10-0132 20663982

[55] Barzi A , Lenz AM , Labonte MJ , Lenz HJ . Molecular pathways: estrogen pathway in colorectal cancer. Clin Cancer Res. 2013;19(21):5842–5848. 23965904 10.1158/1078-0432.CCR-13-0325 PMC3836673

[56] Caiazza F , Ryan EJ , Doherty G , Winter DC , Sheahan K . Estrogen receptors and their implications in colorectal carcinogenesis. Front Oncol. 2015;5:1–9. 25699240 10.3389/fonc.2015.00019 PMC4313613

[57] Ye SB , Cheng YK , Zhang L , Wang XP , Wang L , Lan P . Prognostic value of estrogen receptor‐α and progesterone receptor in curatively resected colorectal cancer: a retrospective analysis with independent validations. BMC Cancer. 2019;19(1):1–7. 31590647 10.1186/s12885-019-5918-4 PMC6781392

[58] Tang W , Liu R , Yan Y , et al. Expression of estrogen receptors and androgen receptor and their clinical significance in gastric cancer. Oncotarget. 2017;8(25):40765–40777. 28388558 10.18632/oncotarget.16582 PMC5522298

[59] Yokozaki H , Takekura N , Takanashi A , Tabuchi J , Haruta R , Tahara E . Estrogen receptors in gastric adenocarcinoma: A retrospective immunohistochemical analysis. Virchows Arch A Pathol Anat Histopathol. 1988;413(4):297–302. 2845639 10.1007/BF00783021

[60] Lee JM , Lee KB , Kim YT , et al. Cervical cancer associated with pregnancy: results of a multicenter retrospective Korean study (KGOG‐1006). Am J Obstet Gynecol. 2008;198(1):92.e1–92.e6. 17905175 10.1016/j.ajog.2007.06.077

[61] Sood A . Cervical cancer diagnosed shortly after pregnancy: prognostic variables and delivery routes. Obstet Gynecol. 2000;95(6):832–838. 10831976 10.1016/s0029-7844(00)00789-4

[62] Hopkins MP , Morley GW . The prognosis and management of cervical cancer associated with pregnancy. Obstet Gynecol. 1992;80(1):9–13. 1318532

[63] Monk BJ , Montz FJ . Invasive cervical cancer complicating intrauterine pregnancy: treatment with radical hysterectomy. Obstet Gynecol. 1992;80(2):199–203. 1635732

[64] Gender Equality Committee of the Executive Yuan. Important gender statistics database: women's Pap smears and screening coverage. [Internet]. [cited 2020 Apr 1]. https://www.gender.ey.gov.tw/gecdb/Stat_Statistics_Field.aspx

[65] Kau YC , Liu FC , Kuo CF , et al. Trend and survival outcome in Taiwan cervical cancer patients: a population‐based study. Medicine. 2019;98(11):e14848. 30882680 10.1097/MD.0000000000014848 PMC6426611

[66] Maggen C , Dierickx D , Lugtenburg P , et al. Obstetric and maternal outcomes in patients diagnosed with Hodgkin lymphoma during pregnancy: a multicentre, retrospective, cohort study. Lancet Haematol. 2019;6(11):e551–e561. 31564649 10.1016/S2352-3026(19)30195-4

[67] Pinnix CC , Osborne EM , Chihara D , et al. Maternal and fetal outcomes after therapy for hodgkin or non‐hodgkin lymphoma diagnosed during pregnancy. JAMA Oncol. 2016;2(8):1065–1069. 27227654 10.1001/jamaoncol.2016.1396 PMC7457973

[68] Registry C , Report A . The cancer registry report in Taiwan in 2015. Health Promot Adm Minist Health Welf. 2018;(December):621.

[69] Nelson RA , Levine AM , Bernstein L . Reproductive factors and risk of intermediate‐ or high‐grade B‐cell non‐Hodgkin's lymphoma in women. J Clin Oncol. 2001;19(5):1381–1387. 11230482 10.1200/JCO.2001.19.5.1381

[70] Chen BK , Yang CY . Parity, age at first birth, and risk of death from non‐Hodgkin's lymphoma: a population‐based cohort study in Taiwan. Int J Environ Res Public Health. 2015;12(8):9131–9140. 26251917 10.3390/ijerph120809131 PMC4555269

[71] Roemer K , Pfreundschuh M . How do estrogens control lymphoma? Blood. 2014;123(13):1980–1981. 24677401 10.1182/blood-2014-02-554691

[72] Ladikou E‐E , Kassi E . The emerging role of estrogen in B cell malignancies. Leuk Lymphoma. 2017;58(3):528–539. 27557932 10.1080/10428194.2016.1213828

[73] Yakimchuk K , Hasni MS , Guan J , Chao MP , Sander B , Okret S . Inhibition of lymphoma vascularization and dissemination by estrogen receptor b agonists. Blood. 2014;123(13):2054–2061. 24470591 10.1182/blood-2013-07-517292

[74] Machado F , Vegas C , Leon J , et al. Ovarian cancer during pregnancy: analysis of 15 cases. Gynecol Oncol. 2007;105(2):446–450. 17292456 10.1016/j.ygyno.2007.01.002

[75] Leiserowitz GS , Xing G , Cress R , Brahmbhatt B , Dalrymple JL , Smith LH . Adnexal masses in pregnancy: how often are they malignant? Gynecol Oncol. 2006;101(2):315–321. 16310839 10.1016/j.ygyno.2005.10.022

[76] Young JL , Cheng WUX , Roffers SD , Howe HL , Correa C , Weinstein R . Ovarian cancer in children and young adults in the United States, 1992–1997. Cancer. 2003;97(S10):2694–2700. 12733134 10.1002/cncr.11351

