# Supplementary material for: Maternal survival of patients with pregnancy‐associated cancers in Taiwan – A national population‐based study
Source: Cancer Med. 2020 Oct 25;9(24):9431–44. doi: 10.1002/cam4.3565 (PMC7774740; doi:10.1002/cam4.3565)
Supplement: Supplementary file 1 — Supplementary Material [file CAM4-9-9431-s001.docx]

**Supplement Table 1.** Survival of patients of different groups (diagnostic year between 2009 and 2015)

|  | N | Crude HR | 95% CI | P-value † | P-value ‡ | HR | 95% CI | P-value † |
| --- | --- | --- | --- | --- | --- | --- | --- | --- |
| **Breast** |  |  |  |  |  |  |  |  |
| Control | 25,582 | 1.00 |  |  |  | 1.00 |  |  |
| Pregnancy | 92 | 2.05 | 1.19-3.53 | <0.01 |  | 1.12 | 0.63-1.98 | 0.70 |
| First year postpartum | 337 | 1.80 | 1.35-2.41 | <0.01 | <0.01 | 1.41 | 1.05-1.92 | 0.03 |
| **Thyroid** |  |  |  |  |  |  |  |  |
| Control | 7,636 | 1.00 |  |  |  | 1.00 |  |  |
| Pregnancy | 33 | 4.89 | 0.67-35.49 | 0.12 |  | 6.67 | 0.88-50.32 | 0.07 |
| First year postpartum | 289 | 0.00 | 0.00-Inf | 1.00 | 0.08 | 0.00 | 0.00-Inf | 1.00 |
| **CRC** |  |  |  |  |  |  |  |  |
| Control | 4,609 | 1 |  |  |  | 1 |  |  |
| Pregnancy | 16 | 1.72 | 0.77-3.84 | 0.18 |  | 0.77 | 0.34-1.72 | 0.52 |
| First year postpartum | 95 | 1.33 | 0.95-1.86 | 0.10 | 0.11 | 1.07 | 0.76-1.52 | 0.69 |
| **Cervix** |  |  |  |  |  |  |  |  |
| Control | 3,154 | 1 |  |  |  | 1 |  |  |
| Pregnancy | 21 | 1.10 | 0.41-2.95 | 0.847 |  | 1.86 | 0.69-5.02 | 0.2195 |
| First year postpartum | 79 | 0.60 | 0.31-1.16 | 0.129 | 0.30 | 0.41 | 0.20-0.84 | 0.0153 |
| **Lymphoma** |  |  |  |  |  |  |  |  |
| Control | 1,747 | 1.00 |  |  |  | 1.00 |  |  |
| Pregnancy | 8 | 0.70 | 0.10-5.01 | 0.73 |  | 1.53 | 0.21-11.02 | 0.67 |
| First year postpartum | 53 | 0.10 | 0.01-0.72 | 0.02 | 0.02 | 0.12 | 0.02-0.88 | 0.04 |
| **Ovary** |  |  |  |  |  |  |  |  |
| Control | 3,220 | 1.00 |  |  |  | 1.00 |  |  |
| Pregnancy | 35 | 0.25 | 0.06-1.00 | 0.05 |  | 0.72 | 0.18-2.92 | 0.65 |
| First year postpartum | 27 | 0.52 | 0.17-1.61 | 0.26 | 0.05 | 1.02 | 0.33-3.21 | 0.97 |
| **Nasopharynx** |  |  |  |  |  |  |  |  |
| Control | 1,121 | 1.00 |  |  |  |  | 1.00 |  |
| Pregnancy | 10 | 0.73 | 0.10-5.20 | 0.75 |  | 0.97 | 0.13-7.00 | 0.97 |
| First year postpartum | 38 | 1.06 | 0.47-2.39 | 0.89 | 0.94 | 0.84 | 0.31-2.32 | 0.74 |
| **Leukemia** |  |  |  |  |  |  |  |  |
| Control | 1,067 | 1.00 |  |  |  |  | 1.00 |  |
| Pregnancy | 17 | 1.44 | 0.74-2.79 | 0.28 |  | 1.61 | 0.82-3.16 | 0.16 |
| First year postpartum | 38 | 1.14 | 0.69-1.88 | 0.61 | 0.49 | 1.29 | 0.77-2.14 | 0.34 |
| **Lung** |  |  |  |  |  |  |  |  |
| Control | 2,927 | 1.00 |  |  |  |  | 1.00 |  |
| Pregnancy | 5 | 2.21 | 0.71-6.87 | 0.17 |  | 2.05 | 0.65-6.39 | 0.22 |
| First year postpartum | 36 | 0.86 | 0.52-1.42 | 0.55 | 0.31 | 1.28 | 0.75-2.19 | 0.37 |
| **Skin** |  |  |  |  |  |  |  |  |
| Control | 994 | 1.00 |  |  |  |  | 1.00 |  |
| Pregnancy | 5 | 0.00 | 0.00-Inf | 1.00 |  | 0.00 | 0.00-Inf | 1.00 |
| First year postpartum | 20 | 0.00 | 0.00-Inf | 1.00 | 0.52 | 0.00 | 0.00-Inf | 1.00 |
| **Stomach** |  |  |  |  |  |  |  |  |
| Control | 1,227 | 1.00 |  |  |  |  | 1.00 |  |
| Pregnancy | 3 | 4.29 | 1.38-13.38 | 0.01 |  | 2.13 | 0.67-6.78 | 0.20 |
| First year postpartum | 22 | 1.78 | 1.10-2.89 | 0.02 | 0.00 | 1.48 | 0.89-2.46 | 0.13 |
| **Primary Brain** |  |  |  |  |  |  |  |  |
| Control | 687 | 1.00 |  |  |  |  | 1.00 |  |
| Pregnancy | 3 | 1.14 | 0.16-8.12 | 0.90 |  | 1.18 | 0.16-8.43 | 0.87 |
| First year postpartum | 12 | 1.57 | 0.70-3.54 | 0.27 | 0.54 | 1.80 | 0.79-4.11 | 0.16 |
| **Others** |  |  |  |  |  |  |  |  |
| Control | 9,979 | 1.00 |  |  |  |  | 1.00 |  |
| Pregnancy | 41 | 1.46 | 0.86-2.47 | 0.16 |  | 2.42 | 1.15-5.11 | 0.02 |
| First year postpartum | 165 | 0.92 | 0.66-1.29 | 0.63 | 0.30 | 0.93 | 0.55-1.60 | 0.80 |

1. Univariate analyses are presented as crude HR at the left column. Multivariate analyses are adjusted for age, diagnostic year, and extend of disease at diagnosis (presented at the right column).
2. Abbreviations: HR, hazard ratio; CI, confidence interval.
3. † Cox proportional hazards; ‡ log-rank test
